# A combination of the activation marker CD86 and the immune checkpoint marker B and T lymphocyte attenuator (BTLA) indicates a putative permissive activation state of B cell subtypes in healthy blood donors independent of age and sex

**DOI:** 10.1186/s12865-020-00343-2

**Published:** 2020-03-20

**Authors:** Susanne Axelsson, Anders Magnuson, Anna Lange, Aseel Alshamari, Elisabeth Hultgren Hörnquist, Olof Hultgren

**Affiliations:** 1 Department of Pathology, Faculty of Medicine and Health, Örebro University, Örebro, Sweden; 2 Clinical Epidemiology and Biostatistics, School of Medical Sciences, Örebro University, Örebro, Sweden; 3 Department of Infectious Diseases, Faculty of Medicine and Health, Örebro University, Örebro, Sweden; 4 Department of Clinical Immunology and Transfusion Medicine, Faculty of Medicine and Health, Örebro University, Örebro, Sweden; 5 Department of Medical Sciences, Faculty of Medicine and Health, Örebro University, Örebro, Sweden

**Keywords:** B cell, B cell subtype, CD86, BTLA

## Abstract

**Background:**

The use of anti-B cell based therapies in immune-mediated diseases targeting general B cell markers or molecules important for B cell function has increased the clinical needs of monitoring B cell subpopulations.

**Results:**

We analyzed the expression profile of cell surface markers CD86 and B and T lymphocyte attenuator (BTLA) in B cell subtypes using flow cytometry, including naïve, transitional, switched memory, non-switched memory and double-negative memory B cells and plasmablasts, and investigated the dependence of age and sex in a healthy adult blood donor population. The switched memory B cell subtype displayed a divergent expression of the markers, with increased CD86 and decreased BTLA as compared to non-switched and double negative memory cells, as well as compared to naïve B cells. Plasmablasts expressed highly increased CD86 compared to all other subtypes and a decreased expression of BTLA compared to naïve cells, but still higher compared to the memory cell populations. Transitional B cells had CD86 and BTLA expression similar to the other naïve cells.

**Conclusions:**

We show divergent expression of CD86 and BTLA in memory cells and plasmablasts compared to naïve B cells independent of age and sex. Furthermore, a similarly divergent difference of expression pattern was seen between the memory cell subtypes, altogether indicating that the combination of CD86 and BTLA might be markers for a permissive activation state. We suggest the combination of CD86 and BTLA expression on B cell subtypes as a potentially important tool in monitoring the status of B cell subtypes before and after treatments influencing the B cell compartment.

## Background

In recent years, there has been an increased number of indications for treating immune-mediated diseases, e.g. multiple sclerosis, rheumatoid arthritis, systemic lupus erythematosus (SLE) and ANCA (anti-neutrophil cytoplasmic antibodies) associated vasculitis, with using biological therapies based on targeted deletion of B cells or interference with B cell development and/or function. The impact on clinical outcome by anti-B cell treatments has sometimes been surprisingly high, even in diseases classically regarded as T cell driven, and despite the survival of the long-lived antibody producing plasma cells (PC). This fact has highlighted other B cell functions besides antibody production, e.g. cytokine production and T cell modulation ability, as important factors in disease progression. The intensified interest in B cell biology may clarify pathogenic mechanism that can lead to the introduction of new B cell targeted therapies. Increased knowledge of differences between B cell subtypes enables more detailed monitoring of the effect of such therapies, and may provide guidance in continued treatment [1, 2]. Furthermore, determining B cell subtypes is of importance in IgG4-related disease [3] and a more detailed description of the status of B cells might be valuable in predicting outcome of vaccination and potentially in making decisions on vaccine regimes [4], and in evaluating activity of chronic viral infections [5].

The expression of CD86 and CD80 on professional antigen presenting cells is of great importance to establish co-stimulation for T lymphocytes via CD28, which might influence activation of T cells or offer T cell help to B cells. Cell surface expression of CD86 was initially demonstrated on human B cells and shown to be quickly upregulated, faster than CD80, following an innate stimulation [6]. The basic expression of CD86 is different on various B cell subtypes, and has been studied in humans using different cell origins, e.g. splenic, tonsillar and peripheral blood B cells. CD86 expression has been suggested to be increased on plasmablasts, being of importance for the production of antibodies, and on memory B cells, compared to naïve B cells where CD86 expression is considered low or undetectable [7–10].

BTLA, together with e.g. Programmed cell death protein-1 (PD-1) and Cytotoxic T lymphocyte–associated antigen 4 (CTLA-4) are designated as immune checkpoint regulators. BTLA (CD272) acts as an inhibitory receptor that mediates its effects upon binding its ligand Herpesvirus entry mediator (HVEM). The effects mediated by BTLA has mostly been studied on T cells where they may inhibit T cell responses, and blocking of BTLA may in turn activate T cells [11]. The role of BTLA signaling in B cells is less well known, although it has been described as an inhibitory co-receptor of the B cell receptor, mediating several inhibitory functions upon HVEM ligation [12, 13]. Few studies have investigated differences in BTLA expression on different B cell subtypes in healthy individuals. There is one study that indicates decreased BTLA expression with age [4]. Although most studies that investigates factors influencing vaccine responses either control for differences between children and young and middle aged adults, or between young and aged adults, sometimes recommendations for vaccination differ also within an adult population. Recently Swedish national recommendations for vaccination for tick-borne encephalitis virus was changed, with an extra dose in the primary immunization of individuals over 50 years of age, a change brought on by an increased incidence of vaccination failures resulting in infection. There are known differences in immune responses between women and men in the clinical context, e.g. efficacy in viral clearance, susceptibility to severe bacterial infections, vaccine responses and incidence of autoimmune diseases. There are several conditions where women display an increased immune response for better for worse [14–16].

To our knowledge, differences with regard to a combination of CD86 and BTLA expressions on B cells or B cell subtypes have not previously been investigated in adult healthy individuals, neither has the dependence of age nor sex for expression of these markers.

We demonstrate a divergent expression, independent of age and sex, of the activation marker CD86 and the immune checkpoint regulator BTLA in plasmablasts and memory B cells compared to naïve B cells, and between different memory B cell subtypes in a healthy adult population.

## Results

### Gating strategy and B cell subtypes

**Fig. 1:**
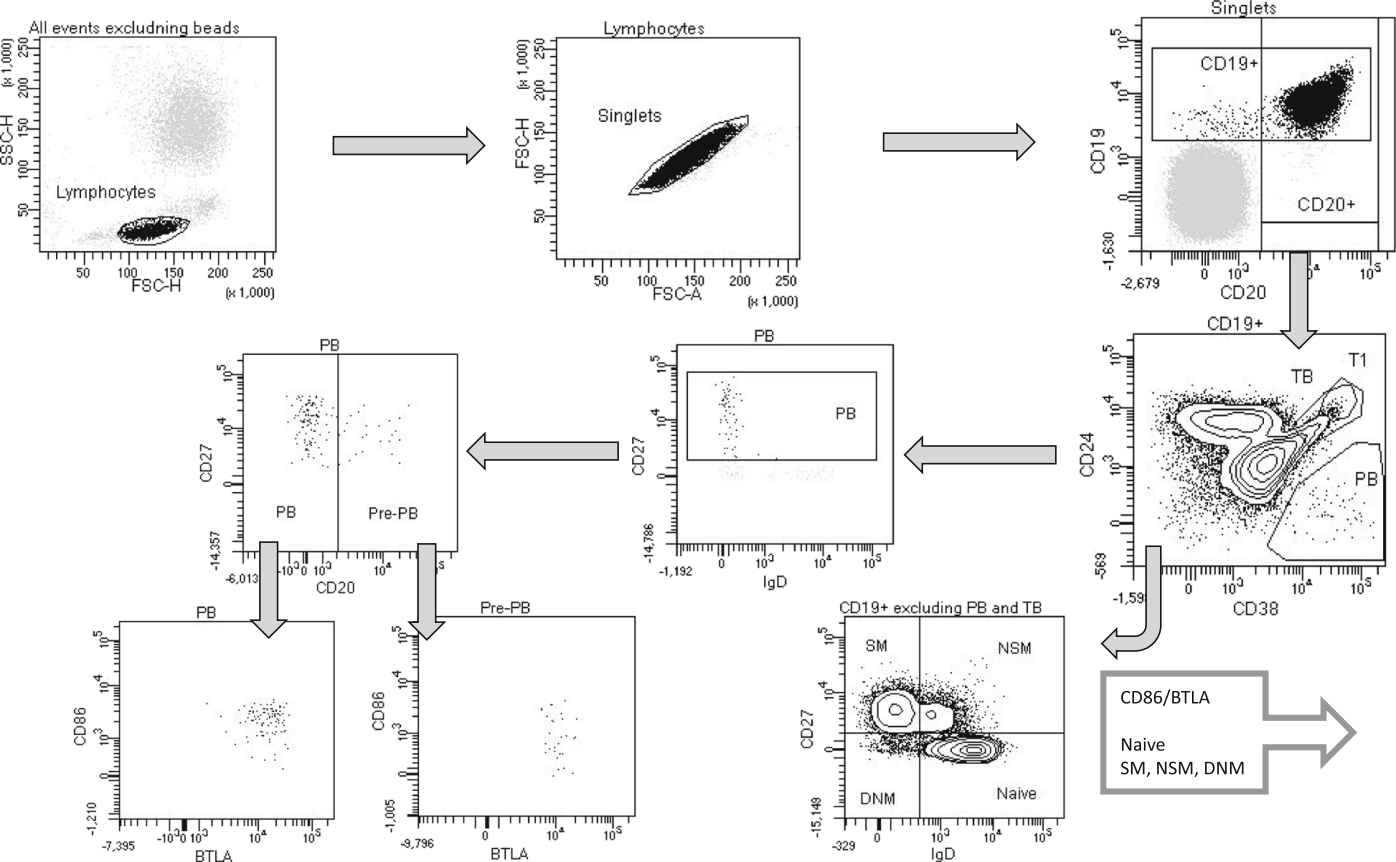
Gating strategy for the different B lymphocyte subtypes from whole blood. The lymphocyte gate was based on FSC and SSC characteristics, followed by a gating of lymphocyte singlets by FSC-H and FSC-A. Plasmablasts (PB) were defined as being CD19 + CD24-CD38++CD27 + CD20-, pre-plasmablasts (pre- PB) as CD19 + CD24-CD38++CD20+ and transitional B cells (TB) as CD19 + CD24++CD38++. The naïve B cells were defined as CD19 + CD27-IgD+ excluding the TB. The switched memory B cells (SM) were defined as CD19 + CD27 + IgD-, the non-switched memory B cells (NSM) were defined as CD19 + CD27 + IgD+, and the double negative memory B cells (DNM) were defined as CD19 + CD27-IgD-. All B cell subtypes were analyzed for CD86 and BTLA expressions, expressed as mean fluorescence intensity (MFI)

The gating strategy is demonstrated in Fig. 1. The lymphocyte population was gated based on FSC and SSC, followed by gating of lymphocyte singlets with FSC-H (Height) and FSC-A (Area). Between 60,000 and 250,000 lymphocytes were analyzed. Thresholds were chosen as follows: CD19 and CD20 based on the presumable population of T cells (CD19 negative and CD20 negative), CD27 and IgD thresholds based on the presumable CD27 negative and IgD positive naïve B lymphocytes on a CD27/IgD plot. CD24^++^ based on a well-defined tip of the B cell population on a CD24/CD38 plot. CD24^−^CD38^++^ gating was based on a well-defined CD24^−^CD38^++^ population. Mean fluorescence intensity (MFI) for BTLA and CD86 were determined for all subpopulations described below.

The naïve B cells were defined as CD19^+^CD27^−^IgD^+^ excluding the CD19^+^CD24^++^CD38^++^ transitional B cells (TB). The switched memory B cells (SM) were defined as CD19^+^CD27^+^IgD^−^, the non-switched memory B cells (NSM) were defined as CD19^+^CD27^+^IgD^+^, and the double negative memory B cells (DNM) were defined as CD19^+^CD27^−^IgD^−^. The transitional B cells (TB) were defined as CD19^+^CD24^++^CD38^++^. Plasmablasts (PB) were defined being CD19^+^CD24^−^CD38^++^CD27^+^CD20^−^. We detected a very small population of CD19^+^CD24^−^CD38^++^ cells being CD20^+^. Since they were also CD27^+^IgD^−^ we had a suspicion that they could be SM that was close to the PB gate, however after back-gating into CD24/CD38 gate that seemed unlikely since the CD20^+^ “PB” were placed clearly in the middle of the PB gate and in accordance with PB they seemed to be rather high in CD27 expression. We defined the CD20^+^ “PB” as pre-plasmablasts (pre-PB) [17] and included them in the analyses of CD86 and BTLA expressions on B cell subtypes.

### Expression of CD86 on B cell subtypes

**Fig. 2:**
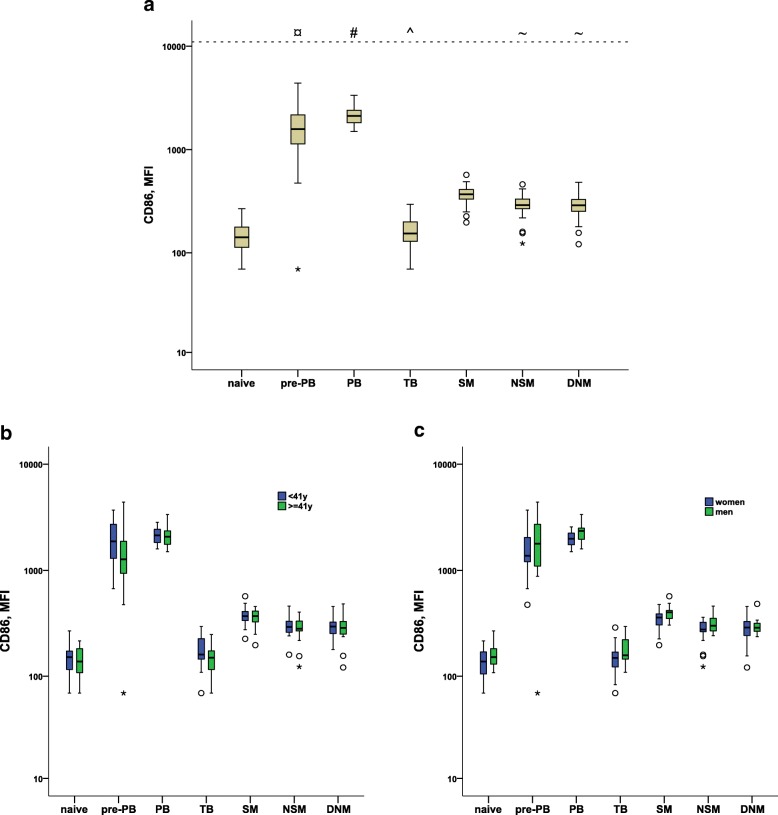
**a** CD86 expression plotted as mean fluorescence intensity (MFI) with boxplots, for B cell subtypes: naïve, pre-plasmablasts (pre-PB), plasmablasts (PB), transitional B cells (TB), switched memory cells (SM), non-switched memory cells (NSM), and double negative memory cells (DNM). **#**PB CD86 expression was significantly higher compared to all other cell subtypes (*P* < 0.0001). **¤**CD86 expression on pre-PB was higher compared to all other celltypes (*p* < 0.0001) except PB. **^**CD86 expression on SM was higher than on naïve (*p* < 0.0001), TB (*p* < 0.0001), NSM (*p* = 0.002) and DNM (*p* < 0.001) B cell subpopulations. **~**Expression on NSM and DNM were higher than on naïve (*p* < 0.0001) and TB (*p* < 0.0001) whereas no significant differences were found between NSM and DNM. All pvalues corrected with Bonferroni-Holm method. Figure is based on results from analyses of samples from 44 blood donors**. b** CD86 expression with regard to age. Figure is based on results from analyses of samples from 22 blood donors < 41 years, and 22 blood donors > = 41 yrs**. c.** CD86 expression with regard to sex. Figure is based on results from analyses of samples from 23 women and 21 men**.** The boxplots shows median, with the upper and lower quartile as box limits and whiskers as min and max if no outliers are present. Outliers are marked with a circle (1,5–3 IQR) or an asterisk (> 3 IQR)

As expected, the naïve B cells displayed a low or absent CD86 expression (Fig. 2a), indicating a non-stimulated state. The transitional B cells (TB), the subgroup of B cells that has newly migrated out of the bone marrow and is joining the other non-stimulated reservoir of naïve cells displayed a similarly low CD86 expression. Since the TB has recently been further sub-grouped into three different stages T1, T2 and T3 based on how recently they have migrated from bone marrow where T1 is the youngest, we checked if the “outer tip” of TB, where we expect to find the T1 cells. This subgroup displayed similar MFI for CD86 (mean 165) as the total TB population (mean 163).

The plasmablast (PB) population, i.e. antibody producing cells circulating in blood before they become non-dividing terminally differentiated plasma cells, which normally take residence in the bone-marrow, displayed significantly highly increased expression of mean CD86 as compared to all other B cell subtypes, as expected (Fig. 2). We analyzed CD86 expression on the CD20^+^ “PB” population, which could potentially be the pre-plasmablasts (pre-PB) described previously [17]. The mean CD86 expression on pre-PB was significantly highly increased compared to all other subtypes except PB.

We also examined CD86 expression on the three most commonly described memory B cell subtypes. All three memory populations displayed statistically significantly increased mean CD86 expression compared to naïve B cells. The switched memory B cells (SM) had significantly higher mean CD86 expression compared to the non-switched memory B cells (NSM) as well as the double negative memory B cells (DNM). There was no significantly difference in mean CD86 expression between the NSM and the DNM.

Interestingly, PB (*p* = 0.016), SM (*p* = 0.016) and NSM (*p* = 0.03) all displayed statistically significant higher mean CD86 expression in men compared to women. The mean MFI of CD86 for PB was 2244 (men) versus 1968 (women), while there were no significant mean differences in CD86 expression in the pre-PB population 1500 vs 1496 (men vs women, *p* = 0.984). The mean MFI for men and women respectively for SM were 392 vs 339, and for NSM 313 vs 267.

In contrary, there were no statistically significant differences in mean CD86 expression on any of the B cell subtype between age groups (< 41 vs ≥41 yrs) or sex and age group interaction effect (Fig. 2b-c).

### Expression of BTLA on B cell subtypes

**Fig. 3:**
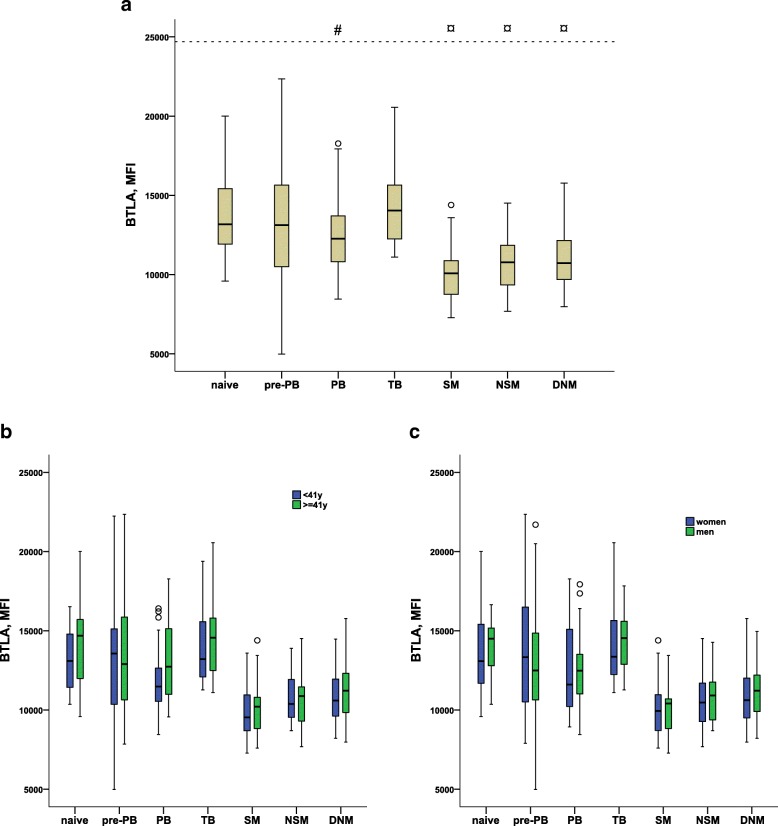
**a** BTLA expression, plotted as mean fluorescence intensity (MFI) with boxplots, for B cell subtypes: naïve, pre-plasmablasts (pre-PB), plasmablasts (PB), transitional B cells (TB), switched memory cells (SM), non-switched memory cells (NSM), and double negative memory cells (DNM). BTLA expression was not significantly different between naïve B cells, TB and pre-PB. **#**PB displayed significantly decreased BTLA expression compared to naïve cells (*p* = 0.002), TB (*p* < 0.0001), and pre-PB (*p* = 0.006). **¤**SM, NSM and DNM displayed lower BTLA expression compared to all other cell types (*p* < 0,00002). SM displayed significantly lower expression compared to NSM (*p* = 0.03) and DNM (*p* = 0.003). BTLA expression on NSM and DNM did not differ significantly. All *p*-values corrected with Bonferroni-Holm method. Figure is based on results from analyses of samples from 44 blood donors. **b** BTLA expression with regard to age. Figure is based on results from analyses of samples from 22 blood donors < 41 years, and 22 blood donors > = 41 yrs**. c** BTLA expression with regard to sex. Figure is based on results from analyses of samples from 23 women and 21 men**.** The boxplots shows median, with the upper and lower quartile as box limits and whiskers as min and max if no outliers are present. Outliers are marked with a circle (1,5–3 IQR)

Due to the possible clinical utility of understanding the biology of BTLA expression, and the hitherto lack of data on the combination of CD86 and BTLA expression, we investigated the expression of BTLA on different B cell subtypes (Fig. 3a). The naïve B cells displayed similar BTLA expression as the TB. Similar to CD86 expression, TB and the putative T1 TB cells displayed similar mean MFI for BTLA (14,246 vs 14,503).

While PB displayed significantly lower BTLA compared to naïve cells, the pre-PB population did not differ compared to naïve cells (Fig. 3). The PB population had significantly lower BTLA expression compared to the pre-PB population.

The memory B cell subtypes SM, NSM and DNM all displayed significantly lower BTLA expression compared to the naïve and PB populations. Similar to the CD86 expression findings, the SM had a significantly different expression pattern as compared to the other two memory cell populations. SM displayed lower expression of BTLA compared to NSM and DNM, whereas we found no statistically significant differences in BTLA expression between NSM and DNM.

In addition, we could not detect any statistically significant differences with regard to BTLA expression due to age (< 41 and ≥ 41 yrs), sex or their interaction effect on any B cell subtype (Fig. 3b-c).

## Discussion

We found a divergent expression of the activation marker CD86 and the immune checkpoint marker BTLA on different B cell subtypes. Naïve and transitional B cells (TB) displayed the lowest CD86 but the highest BTLA cell surface expression. Circulating plasmablasts (PB) and the memory B cell subtypes displayed a more permissive expression pattern, from an activation point of view, with higher CD86 and lower BTLA compared to naïve B cells.

Whereas the interest in B cell functions has been large for a long time mainly due to their ability to become antibody producing plasma cells, the interest for B cell biology has increased substantially during the last decade. The increased use of biologic drugs deleting and/or modulating B cell subtypes has pin-pointed the important roles of B lymphocytes in immune pathology, not only as antibody producing cells to be, but possibly also due to their professional antigen presenting capacity activating T cells, as well as due to e.g. B cell cytokine production. In addition to identifying new properties of B cells, the status of the B cell pool is of interest in immune-modulating therapy, e.g. to show the reoccurrence of B cells or specific B cell subtypes and thereby make decisions on e.g. if a repeated treatment dose is needed.

The low, or negative CD86 expression in the naïve B cell population, including transitional cells is not surprising. As one of the main co-stimulatory molecules, CD86 is usually upregulated following activation of the B cell, which is then able to be further activated and in turn activate T cells. The high BTLA expression on naïve B cells is also logic in the sense that the antigen activation should be the “start button” and until start signal BTLA, along with other inhibitory factors, keeps the B cell in control. One might consider that the earliest naïve newcomers in the circulation, the transitional T1 cells, would have either higher or lower BTLA expression compared to T2, T3 and other naïve B cells. Although our results indicate similar BTLA expression between the TB with the highest CD24 and CD38 expression, containing T1 cell, and the total TB population, i.e. T1–3, this should be further investigated comparing phenotypically well characterized T1–3 cells [18–20]. There are several immune-mediated diseases where treatments affecting the B cell compartment can be considered, and since the TB population is reconstituted early after such treatments, monitoring of its activation pattern, including CD86 and BTLA expression might be useful [7]..

Plasmablasts (PB), the circulating antibody secreting cells (ASC) that is not yet terminally differentiated to plasma cells, are few in healthy individuals. We show that the PB display a more activated phenotype compared to naïve cells. The PB had the highest CD86 expression of all B cell subtypes analyzed in our study, corroborating previously published results [7]. The only B cell population that is close to this pronounced CD86 expression, is the population we suggest are the recently described pre-plasmablasts [17]. We found that pre-PB display significantly higher CD86 compared to naïve and memory B cells. The exact role of the high CD86 expression on PB in humans is not known. In experimental animal models B7–2 (CD86) has been shown to be important for IgG secretion, but it is not clear to what extent the PB CD86 expression influence that outcome [9, 21]). In line with previous published results we found BTLA expression to be significantly decreased on PB as compared to naïve B cells [4], but we also show that the pre-PB had similar expression as naïve cells. The loss of inhibition by BTLA on PB might indicate that BTLA too (similar to CD86) has a role in antibody secretion or maturation of PB. The expression of BTLA changes somewhat slower as compared to CD86, seeing the naïve to pre-PB and to PB in a timeline, which might indicate a later role for BTLA in ASC function. This must however be interpreted with caution since we do not know whether the ASCs are derived from naïve or memory B cells [22]. Therefore, it would be interesting to see future studies investigating BTLA expression on ASCs from those two different origins, and to clarify differences in the developmental route.

We found that memory B cells display increased CD86 expression compared to naïve cells, which has also previously been demonstrated [8]. Furthermore we found that switched memory cells (SM) displayed higher levels of CD86 compared to non-switched memory cells (NSM) and double negative memory cells (DNM). Analyzing CD86 expression on B cell subtypes might be useful knowing that the mutual numbers of and function of SM, NSM and DNM might differ depending on disease activity in rheumatoid arthritis [23]. Our results showing high CD86 in SM are not surprising in the light of previous reports on enhanced responsiveness to anti-CD86 stimulation with regard to production of immunoglobulins in class-switched memory B cell compared to non-class switched memory cells [7]. We also found BTLA to be decreased in all memory cells compared to naïve cells, TB and PB. Once again, we found that SM are the most “activation permissive” memory cell population, with significantly lower BTLA compared to NSM and DNM. Although previously published results on BTLA expression on B cell subtypes are not totally unanimous, our results demonstrating lower expression on memory compared to naïve cells, and PB displaying an intermediate expression are consistent with previous results [4, 5], and likewise are the higher BTLA expression in SM compared to NSM and DNM [4, 13]. The more “activation permissive” state of SM versus NSM and DNM with regard to combined CD86 and BTLA expression might indicate a lower threshold for antigen activation, e.g. meaning less demand of antigen concentrations or other stimuli. One might speculate that the low BTLA expression in SM, together with high CD86, may be one of the factors resulting in rapid differentiation of SM, or at least parts of the SM population, into plasma cells at reactivation together with other factors, e.g. BCR affinity and immunoglobulin tail tyrosine (ITT) motifs.

We did not find any age dependency in BTLA and CD86 expression on B cells. There is, to our knowledge, no previous publications demonstrating any differences for CD86 at the basic expression level on B cells in blood, while there are publications showing differential upregulation of CD80/CD86 on white blood cells following Toll-like receptor (TLR) stimulation in vitro depending on age [24]. There are previous publications demonstrating that the BTLA expression on several B cell subtypes are dependent on age comparing individuals > 65 years with young adults [4], but to our knowledge, no one has previously attempted to find differences within a non-aged adult population.

Like for other immune checkpoint markers PD-1 and CTLA-4, there is a polymorphism of the btla gene that results in less inhibition, and that is associated with rheumatoid arthritis [25]. Several autoimmune diseases, including those with obvious B cell driven pathogenesis, display unequal incidence between sexes, and it possible that this involves differences related to the inhibitory receptors. Therefore we hypothesized that there might be differences dependent on sex in BTLA expression on B cell subtypes. In our relatively small study cohort we could however not find such differences. We did not expect to find any sex dependent differences for CD86 expression. Surprisingly, we found significantly higher CD86 expression on PB, SM and NSM in men compared to women. One might consider that differences are more evident on the cell populations with the highest expression of CD86, but expression on pre-PB, with high CD86 expression, did not differ between sexes. If our finding is true and there actually is a repeatable difference between men and women in CD86 expression on some B cell subtypes, our data indicate that these differences are found in more mature cell sybtypes, PB versus pre-PB, and SM and NSM versus DNM. One previous study describes a clear sex dependent difference in CD80 expression on B cells in neuroinflammatory diseases, independent on disease severity [26]. There is a CD86 polymorphism resulting in higher CD86 expression on B cells [27], and it cannot be excluded that there was an increased frequency of men with that allele in our cohort. It will be interesting to see if our surprising finding will be confirmed by others.

Monitoring not only to the full B cell pool, but also B cell subtypes, is valuable for clinicians in diagnostics and follow-up of treatments directed to diverse niches of the B cell compartment. Studying activating and inhibiting immune markers might add important information in this context and help tailor immune therapy. It is possible that analyses of marker expression before and after lymphocyte stimulation might add important functional aspects of the cell status and therefore being of additional value for decision on choice of treatment. Nevertheless, further studies are needed describing the biology of different B cell subtypes as well as clinical studies where enhanced knowledge of B cell subtypes is taken into account.

The combined examination of CD86 and BTLA as B cell subtype markers enables not only to see temporary changes due to current stimulation, but also to monitor the baseline expression of these markers on different subtypes. We found that the two markers have distinct expression patterns on the different B cell subtypes indicating an activation permissive condition of the cell. Our study also provides evidence that alterations in CD86 and BTLA expression have different kinetics, where CD86 has a capability of quicker adjustment as compared to BTLA in B cell development. It remains to be seen how this combined expression pattern is affected in infectious diseases or in response to treatments of immune-mediated diseases.

## Conclusions

To our knowledge this is the first publication to describe the combination of CD86 and BTLA cell surface markers on different B cell subtypes. We demonstrate a divergent expression of CD86 and BTLA in memory cells and plasmablasts compared to naïve B cells independent of age or sex. Furthermore, a similar difference in expression pattern was seen between the memory cell subtypes, altogether indicating that the combination of CD86 and BTLA might be markers for a permissive activation state. We suggest the combination of CD86 and BTLA expression on B cell subtypes as a potentially important tool in future studies monitoring the status of B cell subtypes before and after treatments influencing B cell compartments.

## Methods

### Subjects and blood samples

Five ml of peripheral blood were collected in EDTA tubes from healthy blood donors at Dept of Clinical Immunology and Transfusion Medicine, Laboratory Medicine, University hospital of Örebro, Örebro, Sweden. The samples were divided into four groups in respect to sex and age (< 41 or ≥ 41 years) as follows: 1) young women (*n* = 11), 2) young men (*n* = 11), 3) old women (*n* = 12), 4) old men (*n* = 10).

### Flow cytometry and antibodies

Flow cytometry was performed on whole blood within 3 h after blood drawing with a lyse-no-wash protocol using BD Trucount™ tubes (Becton Dickinson, BD, San José, CA). All fluorochrome conjugated antibodies were obtained from BD and were mixed according to the manufacturer’s recommendations. The following antibodies were used, Table 1: CD272 (BV421, 564,802, 5 μL), CD86 (BV480, 566,131, 5 μL), IgD (FITC, 555778, 20 μL), CD24 (PE, 555428, 20 μL), CD19 (PerCP-Cy5.5, 561,295, 5 μL), CD27 (PE-Cy7, 560,609, 5 μL), CD38 (APC, 555462, 20 μL), CD20 (APC-H7, 560,734, 5 μL), together with 50 μL Brilliant Stain Buffer (BD, 563794). The antibodies, the Brillliant Stain Buffer and 100 μL of well-mixed anticoagulated whole blood were added to the BD Trucount™ tube, vortexed gently, and incubated for 20 min in the dark at room temperature (RT). Two mL of Pharmlyse (BD), diluted 1:10, was added to the tube, mixed gently and incubated for 10 min in the dark at RT, mixed, and then directly collected in a FACSCanto™ II flow cytometer (BD). The FMO for CD86 and for BTLA were tested showing MFI < 140 (CD86) and < 380 (BTLA) for all B cell subtypes. The settings for forward scatter (FSC) and side scatter (SSC) were optimized to achieve the best separation for lymphocytes, beads and trash. Analyses were performed with FACSDiva Software (BD).

**Table 1:** Antibodies used for flow cytometry analyses

Antigen specificity	Clone name	Fluorochrome	Vendor
CD272 (BTLA)	564,802	BV421	BD^a^
CD86	566,131	BV480	BD
IgD	555,778	FITC	BD
CD24	555,428	PE	BD
CD19	561,295	PerCP-Cy5.5	BD
CD27	560,609	PE-Cy7	BD
CD38	555,462	APC	BD
CD20	560,734	APC-H7	BD

^a^Becton Dickinson

### Statistical methods

SPSS version 22 (IBM, Armonk, NY) was used for all statistical calculations. For each cell type, the BTLA and log_10_ CD86 expression were analysed using two-way ANOVA with sex and age (< 41, ≥41 years) and their interaction term as fixed factors. To compare the outcomes between cell types, a linear mixed model with random intercept was used. Fixed factors were B cell subtypes (naive, pre-PB (pre-plasmablasts), PB (plasmablasts), TB (transitional B cells), SM (switched memory B cells), NSM (non-switched memory B cells), DNM (double-negative memory B cells)), sex and age groups together with all two-way and three-way interaction terms. The reported *p*-values comparing the cell subtypes were adjusted for multiple comparison by the Bonferroni-Holm method [28]. The model residuals were graphically examined and tested by Shapiro-Wilk normality distribution test, and no violation were present which altered the reported statistical significant findings. The statistical significant level was 5%.

## Data Availability

The datasets generated and/or analysed during the current study are not publicly available due to format issue but are available from the corresponding author on reasonable request, in PDF for flow cytometry results and SPSS Statistics 25 data files for figures.

## Abbreviations

ANCA: Anti-neutrophil cytoplasmic antibodies
ASC: Antibody secreting cells
BTLA: B and T lymphocyte attenuator
CD: Cluster of differentiation
CTLA-4: Cytotoxic T lymphocyte–associated antigen 4
DNM: Double-negative memory B cells
FMO: Fluorescense minus one
FSC: Forward scatter
HVEM: Herpesvirus entry mediator
ITT: Immunoglobulin tail tyrosine
MFI: Mean fluorescence intensity
NSM: Non-switched memory B cells
PB: Plasmablasts
PC: Plasma cell
PD-1: Programmed cell death protein-1
SLE: Systemic lupus erythematosus
SM: Switched memory B cells
SSC: Side scatter
TB: Transitional B cells
TLR: Toll-like receptor
